# Supplementary material for: The relationship between visitors’ motivation and landscape preference for the pilgrimage route on the Mount Miaofeng, China
Source: PLoS One. 2024 Dec 2;19(12):e0314194. doi: 10.1371/journal.pone.0314194 (PMC11611186; doi:10.1371/journal.pone.0314194)
Supplement: S1 File — (DOCX) [file pone.0314194.s001.docx]

**Questionnaire of Visitors in the targeted pilgrimage route**

**1.What’s your gender?**

①Male ②Female

**2.Where are you come from?**

Province City

**3.How old are you?**

①Age 14 and younger ②15-24 years old ③25-44 years old

④45 to 64 years old ⑤Age 65 and older

**4.What religion do you follow?**

①Taoism ②Buddhism ③Other religion (Christianity, Islam, etc)

④No religion

**5.How many times have you come to the Mount Miaofeng?**

**6.Who are you traveling with this time?**

①Relatives or friends ②Colleagues or Classmates ③ Other visitors

④To be alone

**7.How do you travel?**

①Individual traveler ②Follow a travel agent ③ Organized by the company

④Follow religious group ⑤Other ways

**8.When do you usually choose to come to Mount Miaofeng? (Multiple options available)**

①Weekends ②Weekdays ③During the Spring Festival (1st to 15th day of 1st lunar month) ④Folk Temple Fair (1st to 15th day of the 4th Lunar month) ⑤Rose Festival (May 25 - June 18) ⑥Red Leaves Festival (mid-September - early November) ⑦Taoist festivals (such as Sanqing Festival and Sanyuan Festival)

**9. Would you like to come to the Mount Miaofeng again?**

①Very reluctant ② Not willing ③Neutral ④Willing ⑤Very willing

**10.Are you mountaineering enthusiasts?**

①Yes ② No

**11.Are you photography enthusiasts?**

①Yes ② No

**12.How long is your tour this time?**

①2 hours ②6 hours ③1 day ④1 to 2 days ⑤2 days or more

**13.What’s your motivation for visiting Mount Miaofeng？**

| No. | Motivation factor | Strongly disagree | Disagree | Neutral | Agree | Strongly agree |
| --- | --- | --- | --- | --- | --- | --- |
| 1 | Express strong religious beliefs |  |  |  |  |  |
| 2 | Worship religious deities |  |  |  |  |  |
| 3 | Fulfill religious obligations |  |  |  |  |  |
| 4 | Seek redemption and blessing to avoid disaster |  |  |  |  |  |
| 5 | Pray for luck and repay wishes with appreciation |  |  |  |  |  |
| 6 | Broaden horizons and gain knowledge |  |  |  |  |  |
| 7 | Satisfy curiosity about religious culture |  |  |  |  |  |
| 8 | Appreciate religious architecture, literature, music and other arts |  |  |  |  |  |
| 9 | Get away from everyday life and relax |  |  |  |  |  |
| 10 | Experience local folklore |  |  |  |  |  |
