# Supplementary material for: The relationship between visitors’ motivation and landscape preference for the pilgrimage route on the Mount Miaofeng, China
Source: PLoS One. 2024 Dec 2;19(12):e0314194. doi: 10.1371/journal.pone.0314194 (PMC11611186; doi:10.1371/journal.pone.0314194)
Supplement: Attachment — Submitted filename: 1 review_mountain pilgrimage_china.docx [file pone.0314194.s003.docx]

The title is descriptive and relevant to the aim of the article, while the abstract effectively summarizes the article's objectives, the methods used, and the main findings.

It is suggested that the authors extend the discussion on the literature review regarding the VEP. It would be interesting to start this discussion with Urry’s concept of the 'tourist gaze.' Typically, every article on visitor-employed photography begins by referencing this concept in one way or another.

The main concern relates to the sample size. It is suggested that the authors extend their explanation for using such a small sample size. For example, the minimum sample size for calculating Cronbach's alpha is generally considered to be at least 100 respondents. As Bonett (Bonett, D. G. (2002). Sample size requirements for testing and estimating coefficient alpha. Journal of educational and behavioral statistics, 27(4), 335-340.) suggested, for scales with a high expected alpha, a smaller sample size might be acceptable, but it is crucial to clearly explain why such a small sample size was deemed sufficient. Another reference might be: <https://wnarifin.github.io/ssc/ssalpha.html>. A similar discussion should be conducted from the perspective of K-means clustering. Overall, the sample size in this part of the research is a weakness of the article, and this concern must be carefully addressed.

The authors should acknowledge as a limitation of their study that visitors were asked to download the Six Foot application. Being aware that they were part of a study might have influenced their behavior as visitors, including the number and types of photos they took. A discussion of this limitation, either in the context of Materials and methods section, either within the literature review where the VEP (Visitor-Employed Photography) topic is addressed, could include relevant references on how user-generated content (UGC), such as travel photos, allows researchers to examine tourists' behavior.
