# Supplementary material for: The relationship between visitors’ motivation and landscape preference for the pilgrimage route on the Mount Miaofeng, China
Source: PLoS One. 2024 Dec 2;19(12):e0314194. doi: 10.1371/journal.pone.0314194 (PMC11611186; doi:10.1371/journal.pone.0314194)
Supplement: Attachment — Submitted filename: Response to Reviewer Comments.docx [file pone.0314194.s004.docx]

Editorial Office

PLOS ONE

Editors

Beijing, October 5^th^, 2024

**Revision for manuscript** **(PONE-D-24-00131 R1)**

**“The relationship between visitors’ motivation and landscape preference for the pilgrimage route on the Mount Miaofeng, China”**

**Dear Editors:**

Thanks a lot for editing our manuscript entitled “The relationship between visitors’ motivation and landscape preference for the pilgrimage route on the Mount Miaofeng, China” (PONE-D-24-00131 R1).

We appreciate the encouragement and thoughtful suggestions from you and the reviewers. We have carefully considered your comments and those of the reviewers, and revised our manuscript accordingly. We provided both a track-change version and an unmarked version for the main manuscript, and prepared a detailed list of our replies (in blue) with line numbers listed.

We hope our revised version is suitable for publication in *PLOS ONE*. Thanks again for your editorial work and we are looking forward to hearing from you soon.

Sincerely,

Feng Xu on behalf of all authors

Response to Reviewer #4:

General comment: The title is descriptive and relevant to the aim of the article, while the abstract effectively summarizes the article's objectives, the methods used, and the main findings.

Comment 1: It is suggested that the authors extend the discussion on the literature review regarding the VEP. It would be interesting to start this discussion with Urry’s concept of the “tourist gaze.” Typically, every article on visitor-employed photography begins by referencing this concept in one way or another.

Response: Thanks for your comment. We have extended the discussion in the literature review regarding the VEP by introducing Urry’s concept of the “tourist gaze.” This addition can be found in the revised manuscript (line 138-143):

*“Consequently, landscape preference embodies the complex interactions between visitors and their surroundings. Urry [46] introduced the concept of the “tourist gaze”, positing that visual perception (i.e., gaze) is central to the tourist experience. Tourists engage with landscapes through subjective lenses, shaping their experiences and preferences at each site. This gaze not only influences what visitors choose to observe but also how they document and interpret their experiences, particularly through photography.”*

46.	Urry J. The Tourist Gaze: Leisure and Travel in Contemporary Societies. Sage Publications; 1990. 202 p.

Additionally, we have extended the discussion in the literature review on VEP by detailing its evolution, as stated in the revised manuscript (line 151-157):

*“Originally, the VEP process involved providing participants with inexpensive disposable cameras and asking them to take a set number of photographs based on personal choice or specific themes. Today, this method often utilizes visitors’ own cameras or mobile devices, making it particularly effective for revealing insights into Chinese visitors’ landscape perceptions**. Photo-taking is an integral social practice among modern Chinese tourists and is generally not perceived as intrusive or difficult, requiring minimal prompting [57].”*

57.	Li C, Li P, Huang X. Liked and Disliked Streetscape Imagery: Relation to Emotional Motivation and Block Distribution From Tourist Bus Visitors. SAGE Open. 2022 Jul 1;12(3):21582440221117129.

Comment 2: The main concern relates to the sample size. It is suggested that the authors extend their explanation for using such a small sample size. For example, the minimum sample size for calculating Cronbach's alpha is generally considered to be at least 100 respondents. As Bonett (Bonett, D. G. (2002). Sample size requirements for testing and estimating coefficient alpha. Journal of educational and behavioral statistics, 27(4), 335-340.) suggested, for scales with a high expected alpha, a smaller sample size might be acceptable, but it is crucial to clearly explain why such a small sample size was deemed sufficient. Another reference might be: https://wnarifin.github.io/ssc/ssalpha.html. A similar discussion should be conducted from the perspective of K-means clustering. Overall, the sample size in this part of the research is a weakness of the article, and this concern must be carefully addressed.

Response: Thanks for your comment. We have provided a more detailed explanation for using a small sample size and included the following statement in the revised manuscript (lines 259-281):

*“Although the sample size is relatively small, it was deemed sufficient for several reasons. First, this study is exploratory (non-confirmatory), so a small sample size is appropriate. And the use of small sample sizes is typical in exploratory VEP research. For instance, Gou and Shibata included 31 participants [5], Li et al. included 30 participants [56], Fefer et al. had only 17 [47], and Markwell included just 15 participants [68]. This suggests that small sample sizes can already obtain enough information to yield effective research outcomes within the VEP field. Second, as Bonett suggested [69], for a single coefficient alpha test, assuming that the Cronbach’s alpha coefficient equals zero in the null hypothesis can yield a minimum sample size of less than 30 to achieve a desired effect size of 0.7. While the general recommendation for sample sizes often suggests at least 100 respondents, Bonett also noted that for scales with a high expected alpha, a smaller sample size might be acceptable. In our study, the Cronbach’s alpha coefficient is 0.811, and the minimum required sample size was calculated to be 34 (using the website: https://wnarifin.github.io/ssc/ssalpha.html, with the minimum acceptable Cronbach’s alpha set at 0.6) [70]. Third, studies have shown that K-means clustering performs better with small sample sizes than with large ones. For example, Kumar and Dhamija applied the K-means clustering algorithm to real-life data with known cluster solutions and found that misclassification percentages increased with sample size, from small samples (n=20 and n=50) to larger samples (n=100, 300, 500, 1000) [71]. Additionally, Henry et al. identified that K-means clustering can produce valid solutions with sample sizes as small as N=20 [72], while another study demonstrated that the standard error of the average percentage of misclassification is lowest with the K-means method under small sample sizes (n=30) [73]. Therefore, including 34 visitors in this research was deemed sufficient.”*

5.	Gou S, Shibata S. Using visitor-employed photography to study the visitor experience on a pilgrimage route – A case study of the Nakahechi Route on the Kumano Kodo pilgrimage network in Japan. J Outdoor Recreat Tour. 2017 Jun 1;18:22–33.

47.	Fefer JP, Hallo JC, Dvorak RG, Brownlee MTJ, Collins RH, Baldwin ED. Pictures of polar bears: Using visitor employed photography to identify experience indicators in the Arctic National Wildlife Refuge. J Environ Manage. 2020 Sep 1;269:110779.

56.	Ye IQ, Hughes K, Walters G, Mkono M. Up close and personal: Using high engagement techniques to study Chinese visitors’ landscape perceptions. Tour Manag Perspect. 2020 Jan 1;33:100629.

68.	Markwell KW. Dimensions of photography in a nature-based tour. Ann Tour Res. 1997 Jan 1;24(1):131–55.

69.	Bonett DG. Sample Size Requirements for Testing and Estimating Coefficient Alpha. J Educ Behav Stat [Internet]. 2002 Dec 1 [cited 2024 Sep 25]; Available from: https://journals.sagepub.com/doi/abs/10.3102/10769986027004335

70.	Arifin W. Sample size calculator (web) [Internet] [Internet]. 2024 [cited 2024 Oct 4]. Available from: http://wnarifin.github.io

71.	Kumar UA, Dhamija Y. Comparative analysis of SOM neural network with K-means clustering algorithm. In: 2010 IEEE International Conference on Management of Innovation & Technology [Internet]. 2010 [cited 2024 Sep 26]. p. 55–9. Available from: https://ieeexplore.ieee.org/abstract/document/5492838

72.	Henry D, Dymnicki AB, Mohatt N, Allen J, Kelly JG. Clustering Methods with Qualitative Data: a Mixed-Methods Approach for Prevention Research with Small Samples. Prev Sci. 2015 Oct 1;16(7):1007–16.

73.	Wahi S, Dash S, Rao A. An Empirical Investigation on Classical Clustering Methods. 2009 Aug 10.

Comment 3: The authors should acknowledge as a limitation of their study that visitors were asked to download the Six Foot application. Being aware that they were part of a study might have influenced their behavior as visitors, including the number and types of photos they took. A discussion of this limitation, either in the context of Materials and methods section, either within the literature review where the VEP (Visitor-Employed Photography) topic is addressed, could include relevant references on how user-generated content (UGC), such as travel photos, allows researchers to examine tourists’ behavior.

Response: Thanks for your comment. We have addressed the limitation regarding visitors being asked to download the Six Foot application in both the literature review and the Materials and Methods sections, and we have cited relevant references on how user-generated content (UGC), such as travel photos, allows researchers to examine tourists’ behaviour.

In the literature review (lines 161-170), we included:

*“Extensive studies utilizing user-generated content (UGC), such as travel photos, allows researchers to examine tourists’ in situ behaviors. For instance, Ye et al. used the VEP method to investigate the on-site perceptions of Chinese tourists in Uluru-Kata Tjuta National Park, showcasing its potential for capturing authentic visitor experiences [56]. Similarly, Nielsen’s study utilized VEP to assess visitors’ perceptions of an urban forest by having volunteers capture images of their most and least favored locations along a trail in a near-urban recreational area in Gothenburg, Sweden [58]. These studies, alongside research on urban streetscapes [57], demonstrate the effectiveness of assessing visual attraction through UGC, further supporting the value of the VEP method in examining tourists’ behaviors.”*

56.	Ye IQ, Hughes K, Walters G, Mkono M. Up close and personal: Using high engagement techniques to study Chinese visitors’ landscape perceptions. Tour Manag Perspect. 2020 Jan 1;33:100629.

57.	Li C, Li P, Huang X. Liked and Disliked Streetscape Imagery: Relation to Emotional Motivation and Block Distribution From Tourist Bus Visitors. SAGE Open. 2022 Jul 1;12(3):21582440221117129.

58.	Nielsen AB, Heyman E, Richnau G. Liked, disliked and unseen forest attributes: Relation to modes of viewing and cognitive constructs. J Environ Manage. 2012 Dec 30;113:456–66.

In the Materials and Methods section (lines 226-231), we stated:

*“However, researchers may question the credibility of prior exposure to the research tool in the VEP method, as awareness of being part of a study might influence visitors’ behavior, such as the number and types of photos taken. Nevertheless, as discussed in the literature review, relevant references on UGC allow researchers to examine tourists’ behaviour and demonstrate the effectiveness of assessing visual preference and examining tourists’ behaviors through VEP.”* In order to minimize disturbance to visitors and make the photos taken reflect their real behavior, two measures have been taken. First, we only inform visitors that they could complete the trip at their own pace and take photos of whatever they wanted, without any right or wrong (lines 286-287). Second, participants worked independently throughout the process without the accompany of investigators (lines 287-289).

Additionally, we explicitly acknowledged this limitation in the conclusion (lines 545-550):

*“Notably, an important limitation of this study is that visitors were asked to download the Six Foot application. Being aware that they were part of a study might have influenced their behavior as visitors, including the number and types of photos they took. This aspect highlights the potential biases introduced by user awareness in user-generated content (UGC), which can affect how tourists’ behaviors are examined. To address this limitation, a control group whose visitors were not aware of the study until they had completed their walk should be included in future research.”*
